# The role of interleukin-6 signalling in pleural infection: observational and genetic analyses

**DOI:** 10.1016/j.ebiom.2025.105887

**Published:** 2025-08-16

**Authors:** Amerikos Argyriou, Alex Robbins, Rachel Scott, Jodie Chalmers, Harrison I.W. Wright, Robin N. Beaumont, Karen T. Elvers, Michael N. Weedon, Nick A. Maskell, David T. Arnold, Fergus W. Hamilton

**Affiliations:** a Academic Respiratory Unit, North Bristol NHS Trust, United Kingdom; b NIHR Biomedical Research Centre, University of Exeter, United Kingdom; c Medicines Discovery Institute, Cardiff University, United Kingdom; d MRC Integrative Epidemiology Unit, University of Bristol, United Kingdom; e Infection Science, North Bristol NHS Trust, United Kingdom

**Keywords:** Pleural infection, Pleural empyema, Interleukin-6, Pleural effusion

## Abstract

**Background:**

Pleural infection is associated with marked local and systemic inflammation leading to significant morbidity. It may be possible to therapeutically augment this response and interleukin-6 is a key signalling cascade in inflammatory pathologies.

**Methods:**

We performed a prospective observational study recruiting patients with pleural effusions secondary to infection and measured interleukin-6 in matched pleural fluid and serum (n = 76). We subsequently performed a large-scale, two sample Mendelian Randomisation study (1601 cases and 830,709 controls), using genetic variation at *IL6R* to proxy the effect of interleukin-6 inhibition on pleural infection and overcome confounding inherent in observational analyses.

**Findings:**

Pleural interleukin-6 levels in infection were 5000-fold higher than matched serum levels (median 72,752 pg/ml vs. 15 pg/ml). Pleural interleukin-6 predicted systemic inflammation (neutrophil count, C- reactive protein), correlated with clinical markers of disease severity (effusion size, pH, glucose), and was strongly associated with length of hospital stay. In Mendelian randomisation analyses, interleukin-6 inhibition was predicted to have a large protective effect on the incidence of infection (OR 0.23; 95% CI 0.14–0.39 per standard deviation decrease in C- reactive protein). The effect size was larger than that seen in COVID-19 and coronary artery disease, where interleukin-6 inhibition has been successful in trials.

**Interpretation:**

Multiple lines of evidence suggest pleural interleukin-6 drives pathology in pleural infection. Targeting interleukin-6 may hold promise and should be considered in randomised trials.

**Funding:**

This study has been funded by the 10.13039/100000002National Institutes of Health and Care Research Bristol Biomedical Research Centre.

**Research in context**

**Evidence before this study**

To identify literature on the role of IL-6 in pleural infection we performed a systematic review using previously published search criteria for pleural disease (PMID: 31248959) from January 2000 to January 2024. Pleural infection search terms were combined with “Interleukin-6 or IL-6 or Interleukin 6 or IL6” which identified 377 studies. All abstracts were screened by two researchers (AA and DTA) and 278 excluded as not relevant, and a further 69 excluded as they did not measure IL-6 in pleural fluid. Of the remaining 14 studies that measured IL-6 in the pleural space, the majority (n = 10) assessed pleural IL-6 role as a diagnostic tool with no link to infection severity or adverse outcomes. The studies identified in the review did not depict a significant correlation between serum and pleural IL-6 levels, but with IL-6 levels in the pleural space being consistently much higher in concentration. In total, 4 studies (n-167 patients) have assessed pleural IL-6 and its association with pleural infection severity. No mendelian randomisation studies were identified on the topic.

**Added value of this study**

To our knowledge, this is the largest prospective study linking pleural IL-6 levels with important clinical outcomes. It has shown that pleural fluid IL-6 levels were dramatically higher and largely independent of serum IL-6 and associated with an increase in length of stay of 6 days for each doubling of IL-6 levels. Using Mendelian randomisation we showed that IL-6 downregulation reduces the incidence of pleural infection.

**Implications of all the available evidence**

There is evidence to suggest that IL-6 downregulation reduces the incidence of pleural infection and represents a promising target for future clinical trials.

## Introduction

Pleural infection (empyema) is a serious condition defined by the collection of infected fluid around the lung. There are approximately 80,000 new cases in the UK and USA every year and given the requirement for extended hospital stays and the potential for thoracic surgery, they represent a significant healthcare cost (over $1.2 billion annually).^1^ Outcomes have seldom changed in the last few decades with a mortality rate of 10% and hospital length of stay of over a fortnight.^2^

It is suspected that ongoing inflammation rather than active infection is a key driver of adverse clinical outcomes in pleural infection. This is supported by: a) limited microbial growth in many samples, or the culture of weakly pathogenic bacteria only^3^^,^^4^; b) effective antimicrobial levels being achieved in the pleural space with standard dosing^5^; c) the relative effectiveness of host-directed therapy in randomised trials (e.g., DNAse and streptokinase in MIST2).^6^ As such, randomised trials of steroids have been performed in parapneumonic effusions with a positive signal in the paediatric population but with no full-scale trials in adults.^7^^,^^8^ A potential driver of ongoing inflammation in pleural infection is interleukin-6 (IL-6).

IL-6 is a cytokine pivotal to an effective immune response. It is secreted mainly by immune, adipocyte and musculoskeletal cell-types.^9^ IL-6 must bind to the IL-6 receptor (IL-6R) protein and then subsequently to transducer glycoprotein 130 (gp130) for it to effect downstream signalling. IL-6/IL-6R/gp130 forms a transmembrane hexameric complex which can then activate the MAP kinase, JAK/STAT and phosphatidylinositol 3-kinase/Akt pathways.^9^ Importantly, IL-6R is found as a membrane-bound protein (mIL-6R) upon hepatocytes and haematopoietic cell-types, where *cis* signalling occurs, but also as a soluble protein (sIL-6R) found in all fluid compartments of the body. As gp130 exists in all cell types and sIL-6R is found in serum, IL-6 is theoretically able to stimulate all cell types in the body via *trans* signalling. With this mechanism of action, IL-6 exerts an effect upon the integrated immune system through the release of acute-phase proteins (notably C-reactive protein [CRP]), the maturation and differentiation of T-cells, as well as immunoglobulin synthesis.^9^

Although IL-6 levels have long been established as predictive of outcome in many infections,^10^ the most compelling evidence of a causal link between IL-6 and outcomes is depicted in severe COVID-19, where Mendelian randomisation studies identified a protective effect of reduced IL-6 signalling^11^ before this was confirmed by randomised trials.^12^ Other genetic studies have shown that higher levels of IL-6 are predicted to causally increase the odds of sepsis and sepsis-related death, as well as incur an increased risk of tuberculosis infection^13^^,^^14^; conversely, they are likely to be protective in pneumonia.^11^ This data is consistent with registry and trial data of IL-6 inhibition,^15^ which suggests that IL-6 inhibition in autoinflammatory conditions increases the risk of infection, but that targeting IL-6 in severe infection might have benefit.

Given the evidence supporting adjunctive anti-IL-6 therapy in severe COVID-19, we sought to review evidence that targeting IL-6 might have a role in pleural infection. Specifically, this study aimed to answer two key questions: 1) What are the relationships between pleural fluid IL-6, serum IL-6, and key clinical outcomes (e.g., length of hospital stay) in patients presenting with pleural infection? 2) Does genetically-predicted variation in IL-6 receptor signalling causally influence the risk of developing pleural infection?

## Methods

### Observational analyses

#### Patients

Between 2009 and 2016, patients presenting to a UK tertiary pleural service with undiagnosed pleural effusions were prospectively recruited to an observational study (Investigation of Pleural Disease and Improving Patient Pathway^16^). All had routine serum and pleural fluid analysis including a full blood count, serum C-reactive protein (CRP), and pleural fluid pH, glucose and lactate dehydrogenase (LDH). At the time of pleural fluid sampling, pleural ultrasound was performed by a physician who was at least Level 1 British Thoracic Society (BTS) ultrasound-trained (or equivalent). The presence of loculations was documented. Effusion size was split into 3 categories depending on radiological size of the effusion on admission chest radiograph: 1) < 25% hemi-thorax; 2) 25–50% hemi-thorax; 3) > 50% hemi-thorax.

Patients gave consent for storage for future analysis of their baseline pleural fluid and serum samples. Samples were stored in a −70 °C freezer. Blood samples consisted of centrifuged plasma stored in ethylene diamine tetra-acetate (EDTA) anticoagulant tubes, while pleural fluid was stored as a supernatant in EDTA tubes, both in 500 μL aliquots.

Patients were treated as per standard care and followed up at 12 months to ascertain the final diagnosis of their pleural effusion. The final diagnosis was decided by two independent respiratory consultants based on pre-specified criteria. Effusions found to be secondary to infection were categorised using BTS criteria into simple parapneumonic (SPE), complex parapneumonic (CPE) and pleural infection.^17^

#### Sample analysis

Serum and pleural samples were thawed and assayed on the same day in duplicate with a commercial Human IL-6 uncoated ELISA Kit (Invitrogen ref. 88-7066-88). The specific commercial kit 2-day protocol was followed for all assays, using a Corning Costar 96-well plate. A BMG LABTECH plate reader and software were used for all quantitative analysis, following Invitrogen manufacturer parameters and settings. A standard 4-Parameter fitted regression curve was plotted for all samples, with an R^2^ value for the curve reading >0.99 for all plates.

#### Statistics

Descriptive and inferential analysis of data was conducted using R 4.4.0 (R Foundation for Statistical Computing, Vienna). All values for continuous data are given as median with interquartile range (IQR), while discrete data is presented as value with percentage. Statistical significance was set at the 95% confidence interval and presented as p-value with confidence intervals, where appropriate.

We estimated the relationship between pleural IL-6, serum IL-6 and other important biomarkers of pleural infection using Pearson's correlation, visualized in a correlation plot. Subsequently, we assessed the effect of pleural IL-6 on key clinical and radiological outcomes: presence of pleural loculations, requirement for rescue therapy (fibrinolytics or thoracic surgery), and hospital length of stay. Linear or logistic regression was performed using the built-in functions in R, adjusting for age and sex only in one model while covariates for a second, adjusted model in the observational analysis were selected based on their known clinical relevance to pleural infection outcomes and potential confounding effects identified in the literature. These included patient age, sex, pleural fluid protein, LDH, glucose, and pH levels, and serum C-reactive protein levels.

### Mendelian randomisation

To assess whether IL-6 activity had a causal role in infection pathogenesis we used Mendelian randomisation (MR).^18^ MR is an approach that uses the random allocation of alleles at conception to generate estimates of the causal effects of exposures on an outcome. It has three core assumptions which are required for any instrumental variable approach. Firstly, that the genetic variation is associated with the exposure (relevance); secondly, that there are no causes for the genetic variation that influence the outcome except through the exposures (exchangeability); thirdly, that the genetic variation acts through the exposure only (exclusion restriction).^19^

In this analysis, we aimed to use the exposure of IL-6 signalling, identifying genetic variation in and around the gene *IL6R,* that encodes for IL-6R, the sole receptor for IL-6. This approach has been widely used in MR studies before,^11^^,^^13^^,^^14^ and analyses using these methods have closely matched both positive and negative randomised controlled trial data.^12^^,^^20^ It is important to note that IL-6 signalling is complex: it includes both classical (*cis*) signalling, where IL-6 binds to mIL-6R on cells such as hepatocytes, and *trans* signalling, where IL-6 binds to sIL-6R in plasma and can, in principle, stimulate all cells.

Our approach was to identify genetic variants in close proximity to the IL-6R gene (within 300 kb) associated with increased serum levels of IL-6R and decreased levels of CRP. Increased sIL-6R in serum acts as a buffer for IL-6, genetic variants that increase sIL-6R are predicted to reduce classical signalling. Although the mechanism of action of this genetic mutation is unknown for all variants, for one major variant (rs2228145), the C allele is known to lead to increased splicing of mIL-6R into the plasma and thereby lead to reduced classical signalling.^21^ Carriers of this allele therefore have reduced CRP and fibrinogen as well as a reduced risk of cardiovascular disease.^22^

To derive the IL-6R instrumental variable (IV) we first took SNPs that were associated with an increased IL-6R serum level in 54,219 participants from the UKB Pharma Proteomics Project (UKB-PPP) cohort (p < 5 × 10^−8^) and decreased CRP in the UK Biobank/CHARGE consortium meta-analysis, both of these studies are from European ancestries.^23^^,^^24^ SNPs within 300 kb of the IL-6R gene were selected and were then clumped together with an r^2^ threshold of 0.1 in 1000 Genomes European ancestry subset.^25^ SNPs were then weighted by their effect on CRP and only those with an F-statistic >10 included in the IV. This yielded an instrumental variable (IV) comprised of 44 independent SNPs (Supplementary Table S1). Sensitivity analyses were performed restricting to only SNPs that were pQTLs (p < 5 × 10^−8^) for CRP in addition to IL-6R (n = 35) (Supplementary Table S2).

For our infection outcome, we used the recent meta-analysis of infection from FinnGen^26^ and UK Biobank^27^ (https://public-metaresults-fg-ukbb.finngen.fi/pheno/J10_PYOTHORAX). Both are large, population-scale cohorts, with around 400,000 participants each of European ancestry. Infection was defined using ICD-10 coding (pyothorax–J86). This code, appearing in any diagnostic position, has been previously validated for the clinical diagnosis of pleural infection.^28^ In total we included 420 cases in UK Biobank (420,111 controls) and 1201 cases in FinnGen (410,598 controls). Details on the inclusion criteria, genetic quality control and GWAS pipeline are available at the relevant websites.^26^^,^^29^

We extracted the relevant beta coefficients and associated standard errors from this GWAS and harmonised the data with the exposure dataset. For our primary analyses we calculated MR effects using the Wald ratio for each SNP, we then used fixed effects inverse variance weighted MR across SNPs to generate a summary estimate. As our exposure is weighted on CRP, the effect estimate is per change in natural log CRP as this was the scale of the exposure GWAS.^23^ To aid comparison, we also performed MR using the same exposure on other conditions where IL-6 signalling is known to be effective or hypothesised as a potential target: cardiovascular disease, COVID-19, and rheumatoid arthritis. We report all estimates per decrease in CRP, reflecting the effect of reduced IL-6 signalling. Estimates were extracted from our recent analysis.^13^

As sensitivity analyses, we a) ran MR using more strictly independent SNPs (r^2^ threshold 0.01) yielding an IV with 12 independent SNPs (Supplementary Table S3); b) ran leave-one out analyses; c) excluded outliers using Radial-MR. We also report results from MR-Egger to assess the risk of horizontal pleiotropy affecting the validity of our conclusions. MR-Egger regression was used to estimate directional pleiotropy via its intercept term; a non-zero intercept (p < 0.05) suggests the presence of directional pleiotropy. Additionally, we performed a Steiger test on our results to support the assertion that the causal direction was from exposure to outcome and provide evidence that these results are not subject to reverse causality. We additionally performed a phenome wide association study (PheWAS) on all SNPs included in the instrumental variable using the Integrated Epidemiology Unit (IEU) GWAS catalogue and ieugwasr R package to assess association with other biomarkers and conditions that may suggest pleiotropy. These sensitivity analyses are driven by recent simulation and empirical work on *cis-*MR specifically.^30^

Because UKB contributed to both our exposure GWAS of IL-6R (n = 54,219) and our outcome GWAS meta-analysis with Finngen (total n = 830,709) there was a maximum potential sample overlap of 6.5%. To confirm that the associations between IL6R signalling and empyema were robust and not merely a product of ‘Winner's curse’ and weak instrument bias we derived the exposure IV in an independent cohort with no sample overlap. deCODE is an Icelandic population-based study with proteomic data available for 36,000 individuals. GWAS summary statistics are available and were used to derive the IL-6R IV as previously described. This yielded 14 independent SNPs (Supplementary Table S4). Inverse variance weighted MR was then performed.

### Ethics

The Investigation of Pleural Disease and Improving Patient Pathway study was performed under ethical approval (08/H0102/11). Ethics approval for the UK Biobank study was obtained from the Northwest Centre for Research Ethics Committee (11/NW/0382). The FinnGen study is approved by the THL (approval number THL/2031/6.02.00/2017, amendments THL/1101/5.05.00/2017, THL/341/6.02.00/2018, THL/2222/6.02.00/2018, THL/283/6.02.00/2019 and THL/1721/5.05.00/2019), the Digital and Population Data Service Agency (VRK43431/2017-3, VRK/6909/2018-3 and VRK/4415/2019-3), the Social Insurance Institution (KELA) (KELA 58/522/2017, KELA 131/522/2018, KELA 70/522/2019 and KELA 98/522/2019) and Statistics Finland (TK-53-1041-17).

### Role of funders

The funders had no role in the study design, analysis or manuscript preparation.

## Results

### Observational study of serum and pleural IL-6 levels

Between 2009 and 2016, 1005 consecutive patients with undiagnosed effusions were recruited to the prospective study and had baseline biological samples collected and stored. We identified 86 cases secondary to infection with stored biological samples which were subsequently assayed for IL-6 levels. The demographics, baseline biochemistry and IL-6 levels are shown in Table 1.

**Table 1:** Demographics and clinical data on the observational cohort.

Characteristic	N = 86
Age (years)	62^a^ (44, 76)
Female sex	30 (35%)
1-year mortality	12 (14%)^b^
Serum IL-6 (pg/ml)	15 (10, 24)^a^
Pleural IL-6 (pg/ml)	72,752 (28,220, 115,563)^a^
Pleural fluid pH	7.29 (6.95, 7.40)^a^
Pleural loculations on ultrasound	53 (62%)^b^
Serum CRP (mg/L)	132 (71, 197)^a^
Length of hospital stay (days)	14 (5, 21)^a^

Median pleural IL-6 levels were approximately 5000 times higher (72,752 pg/ml, IQR 28,220–115,563) than serum levels (15 pg/ml; IQR 10–24, p = 1 × 10^−15^).

a Median (Interquartile range (IQR)).

b n (%).

**Fig. 1:**
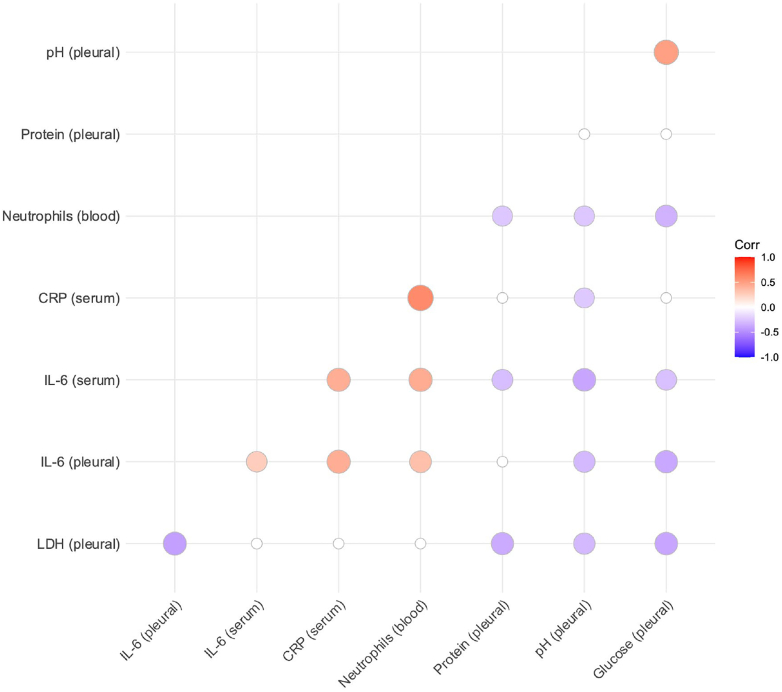
Correlation between blood and serum biomarkers in our observational analyses. Only nominally significant associations (p < 0.05) shown. Darker spots indicated stronger correlation, (red = positive correlation, purple = negative correlation).

We subsequently assessed the relationship between other pleural and blood-based biomarkers and IL-6 (Fig. 1). Pleural IL-6 was moderately correlated with higher blood neutrophil count (Pearson's R 0.43, p < 0.001), lower pleural glucose (Pearson's R −0.37, p = 0.002), and lower pleural pH (Pearson's R −0.30, p = 0.003). There was weak evidence of an association with higher serum IL-6 and lower pleural protein (Pearson's R −0.28, p = 0.03).

We evaluated whether pleural or serum IL-6 was associated with clinically relevant outcomes such as the presence of pleural loculations, requirement for rescue therapy (fibrinolytics or thoracic surgery), or hospital length of stay. Analyses were performed adjusting for age and sex only or additionally accounting for other routinely tested biomarkers (protein, LDH, glucose, pH, and CRP) (Table 2).

**Table 2:** Association between logged pleural or serum IL-6 and key clinical outcomes.

Outcome	Regression strategy (estimate, 95% CI, p value)	
	Age & sex only	Fully adjusted^a^
Pleural IL-6 (log)		
Loculations	2.32 (1.36, 3.97), p = 0.002	3.56 (1.07, 11.79), p = 0.038
Requirement for intrapleural fibrinolytics or surgery	2.11 (1.14, 3.90), p = 0.017	1.75 (0.67, 4.63), p = 0.255
Hospital length of Stay	5.64 (3.49, 7.79), p < 0.001	4.69 (1.36, 8.02), p = 0.007
Serum IL-6 (log)		
Loculations	2.12 (1.15, 3.91), p = 0.016	3.95 (0.82, 19.03), p = 0.087
Requirement for intrapleural fibrinolytics or surgery	1.65 (0.99, 2.77), p = 0.055	0.79 (0.29, 2.16), p = 0.649
Hospital length of Stay	2.84 (0.37, 5.31), p = 0.027	−1.09 (−5.17, 2.99), p = 0.603

Estimates generated by linear regression for linear outcomes (beta), and logistic regression for binary outcomes (odds ratio).

a Full adjustment included age, sex, log (serum IL-6), pleural fluid LDH, pleural fluid glucose, pleural fluid pH, and serum C-reactive protein.

Comparison of Akaike Information Criterion (AIC) values indicated that the fully adjusted models consistently provided a better model fit (lower AIC) compared to the corresponding age- and sex-adjusted models across all outcomes (e.g., AIC for the fully adjusted loculations model with pleural IL-6 was 66.6 vs. 111.1 for the age/sex only model; AIC for the length of stay model with pleural IL-6 was 519 vs. 644.1). To assess calibration of the fully adjusted logistic regression models, we performed the Hosmer–Lemeshow test; this suggested adequate model calibration for predicting loculations (p = 0.71 for pleural IL-6 model; p = 0.39 for serum IL-6 model) and rescue therapy (p = 0.85 for pleural IL-6 model; p = 0.82 for serum IL-6 model), with all p-values >0.05 indicating no significant lack of fit. Finally, we examined multicollinearity among predictors in the fully adjusted models using Variance Inflation Factors (VIFs); all VIFs were low (maximum VIF observed was 2.3), suggesting that multicollinearity was not a significant concern influencing the model estimates.

**Fig. 2:**
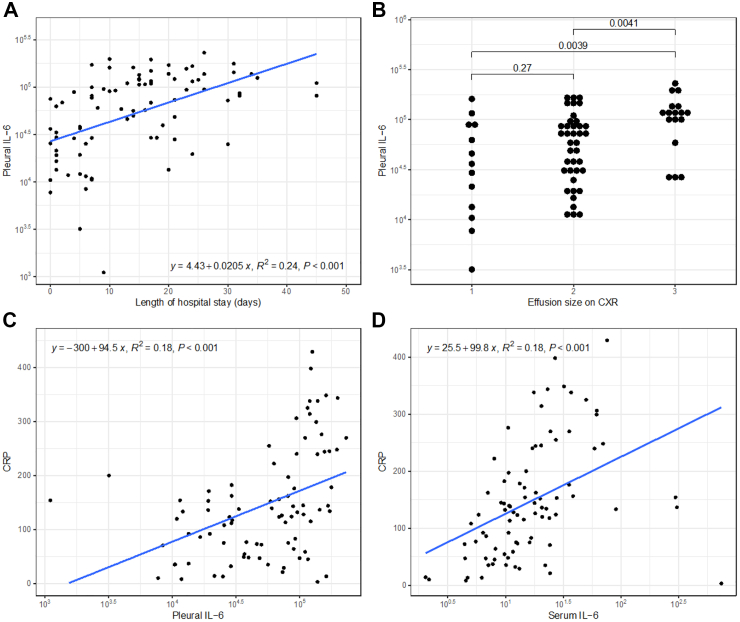
**Panel A** shows the association between pleural IL-6 (y-axis) and length of hospital stay (x-axis). **Panel B** shows the association between pleural IL-6 (y-axis) and effusion size, graded from 1 to 3 [1) < 25% hemi-thorax; 2) 25–50% hemi-thorax; 3) > 50% hemi-thorax]. **Panel C** shows the association between pleural IL-6 (x-axis) and CRP (y-axis), while **panel D** shows the association between serum IL-6 (x-axis) and CRP (y-axis). The blue lines represent the fitted regression line and 95% confidence interval.

In this analysis, both pleural and serum IL-6 predicted key clinical outcomes. Length of stay increased by 5.64 days (95% CI 3.49, 7.79, p = 1.81 × 10^−6^) per each increase in log pleural IL-6 (Fig. 2A). In general, associations were stronger with pleural IL-6 compared to serum IL-6 and were more statistically robust. Finally, we tested whether pleural IL-6 was associated with the size of the effusion on chest radiograph finding that larger effusion had greater pleural IL-6 levels compared to small effusions on chest x-ray (Fig. 2B).

Importantly, the correlation between serum and pleural IL-6 was weak (Pearson's R = 0.15, p = 0.02), but both were strongly associated with serum CRP (Fig. 2C and D). In a multivariable model using age and sex, both pleural IL-6 and serum IL-6 independently predicted CRP levels, with almost exactly the same effect size and proportion of variance explained (increase in CRP with each log increase in pleural IL-6 35.4 mg/l, 95% CI 14.9–55.9; serum IL-6 35.9 mg/l; 16–55.9; variance explained 13%; 14%) (Fig. 2C and D).

In summary, our observational analyses identified that IL-6 was extremely elevated in pleural fluid and was associated with numerous clinical features of severity of pleural infection. In contrast, serum levels of IL-6 were much lower, and often only marginally higher than the normal range^31^; associations with clinical outcomes were weaker. However, both measures independently predicted CRP.

### Mendelian randomisation

**Fig. 3:**
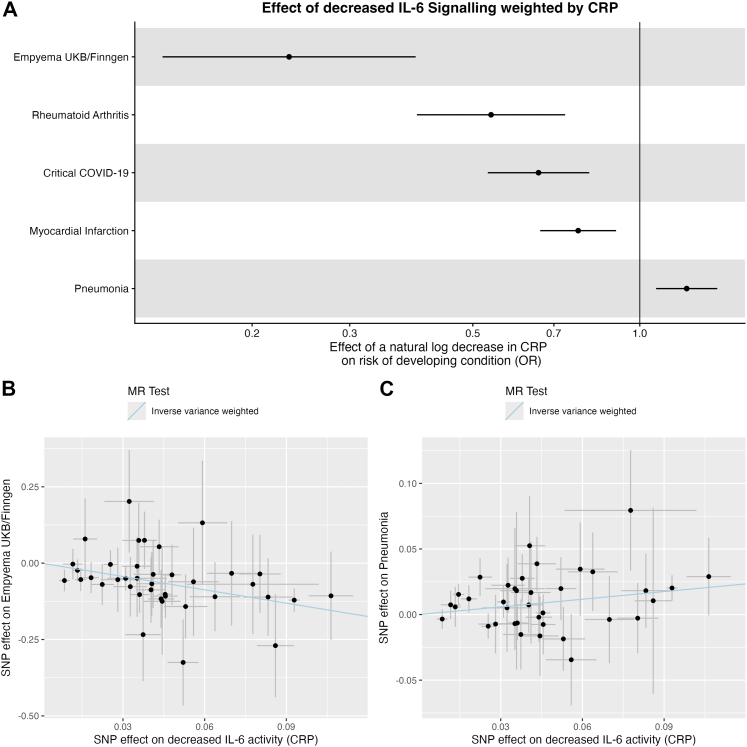
**Panel A** represents the odds ratio for developing each outcome per unit decrease in IL-6 activity (weighted by CRP). Odds ratios under one should be interpreted as protective; i.e. IL-6 inhibition reduces the odds of pleural infection. **Panel B** shows each individual SNP effect on CRP as a marker of IL-6 signalling, and on pleural infection. **Panel C** shows each individual SNP effect on CRP and pneumonia. As we are instrumenting IL-6 inhibition, these SNP effects are the negative effect of each SNP on CRP.

To explore whether IL-6 is causal in infection, or simply correlated with disease, we used Mendelian randomisation. In inverse variance weighted analysis, proxied IL-6 inhibition was associated with greatly decreased odds of developing pleural infection (OR 0.23, 95% CI 0.14–0.39 per standard deviation decrease in log CRP) (Fig. 3A and B). This was a larger magnitude of effect than that seen in other diseases where IL-6 signalling is implicated: rheumatoid arthritis (OR 0.54, 95% CI 0.40–0.73); critical COVID-19 (OR 0.66, 95% CI 0.53–0.81); myocardial infarction (OR 0.77, 95% CI 0.66–0.91). Conversely, and in line with previous literature, proxied IL-6 inhibition was associated with an increased odds of developing pneumonia (OR 1.21, 95% CI 1.07–1.38) (Fig. 3C & Supplementary Table S5).

These results were robust to multiple sensitivity analyses including increased stringency of SNP independence, with R^2^ < 0.01 (OR for pleural infection 0.26, 95% CI 0.13–0.54) (Supplementary Table S6). Restriction to SNPs that were genome wide significant (p < 5 × 10^−8^) pQTLs for both IL6R and CRP independently (OR for pleural infection 0.24 95% CI 0.14–043) (Supplementary Table S7). Removal of outlier SNPs by Radial MR (OR for pleural infection 0.23 95% CI 01.6–0.34) and the removal of any single SNP in leave one out analyses, with removal of rs2228145 leading to the greatest decrease in precision (OR 0.21, 95% CI 0.10–0.42) but no change in effect estimates (Supplementary Tables S8 and S9).

MR-Egger analysis was performed and did not demonstrate significant evidence of horizontal pleiotropy (Pleural infection Egger intercept −0.019 Standard Error 0.019, p = 0.31) (Supplementary Table S10). Steiger testing was supportive of the conclusion that SNPs included in the IV were affecting IL-6R signalling and this exposure was responsible for the affect on the risk of empyema development (Supplementary Table S11).

Additionally, results were replicated using a non-overlapping population from the deCODE study to derive the IL-6R IV and this confirmed a similar magnitude and direction of association with risk of empyema (OR 0.26 95% CI 0.13–0.51) and pneumonia (OR 1.19 95% CI 1.00–1.42) (Supplementary Table S12).

Separately a PheWAS analysis of the SNPs included in the initial IV demonstrated no genome wide significant associations with BMI or cardiometabolic diagnoses, however, rs2228145 and rs9426831 did show an association with apolipoprotein A-I levels, albeit far weaker than the association with CRP levels (Supplementary Table S13). These are downstream of IL-6 signalling which is recognised to affect lipid biology.^32^

## Discussion

Pleural infection remains a challenging condition to treat. In this paper, we provide multiple lines of evidence that IL-6 is an important component of the inflammatory cascade and represents a potential therapeutic target. Firstly, we showed that pleural fluid IL-6 levels were dramatically higher and largely independent of serum IL-6, supporting a compartmentalised, local inflammatory response, although serum IL-6 levels still remain useful as a non-invasive marker Secondly, we showed that pleural IL-6 independently predicted serum CRP, a marker of severe disease, as well as other key pleural biochemical measures. Thirdly, we found a large and clinically meaningful effect of pleural IL-6 on clinical outcomes, including an increase in length of stay of 6 days for each doubling of IL-6 levels. Further to this, we highlighted that the increase of IL-6 in the pleural space occurs independently to a visible rise in systemic IL-6 levels. Finally, we used Mendelian randomisation to show that IL-6 downregulation reduces the incidence of pleural infection. Importantly, our MR analysis revealed contrasting effects of genetically proxied IL-6R inhibition on pleural infection (reduced risk) vs. pneumonia (increased risk, OR 1.21, 95% CI 1.07–1.38). This divergence underscores the complex role of IL-6 in host defence, which may differ significantly between anatomical compartments and infection types. While excessive IL-6 activity in the pleural space may drive damaging inflammation associated with poor outcomes in pleural infection, IL-6 likely plays a crucial role in early pulmonary immune responses, such as neutrophil recruitment, necessary for controlling primary lung pathogens. Inhibition of this pathway could potentially impair bacterial clearance in the lung parenchyma, leading to increased pneumonia susceptibility or severity. This highlights that therapeutic modulation of IL-6 signalling must consider the specific infectious context and potential trade-offs. The lack of any significant rise in IL-6 seen systemically in both our sample and our review, suggests the focus of inflammation occurs solely in the pleural space.

Traditional pleural fluid biomarkers, such as pH and LDH, are used to indicate the need for chest drain insertion but have never been prospectively assessed and do not have prognostic value beyond this in adults.^33^ Recent work has sought to identify biomarkers that can predict important clinical outcomes as the disease can be hard to manage and prognosticate. In an observational study of 93 patients with pleural effusions secondary to infection, soluble urokinase-type plasminogen activator receptor (suPAR) was identified as a predictor for fibrinolytics and referral to thoracic surgery, outperforming a combination of pH, LDH and glucose.^16^ Bedawi et al. analysed 214 pleural samples from patients with pleural infection and found that higher plasminogen activator inhibitor 1 (PAI-1) levels were associated with increased length of stay and were found to be an independent predictor of mortality at 12 months.^34^ There is limited evidence from a study of 30 patients that pleural cell free mitochondrial and nuclear DNA also correlate with days of admission.^35^ We identified 4 studies, totalling 167 patients, that measured IL-6 levels in infected pleural fluid and correlated them with clinical outcomes.^36^, ^37^, ^38^, ^39^ The largest study (n = 57) performed by Chiu and colleagues, measured pleural IL-6 levels (not serum) alongside other pro-inflammatory cytokines (tumour necrosis factor-α (TNF-α), interleukin-1β (IL-1β), and IL-6, tissue-type plasminogen activator (tPA) and plasminogen activator inhibitor type 1 (PAI-1)) in a paediatric population presenting with parapneumonic effusions. They demonstrated that pleural IL-6 levels rose as the complexity of effusion increased on ultrasound, and patients with higher IL-6 levels were more likely to require pleural intervention, although the study design could not assess causality of IL-6 upregulation and clinical outcomes.

In this study, pleural levels have been found to be higher than serum levels, although previous ratios (e.g., 10-fold in parapneumonic effusion^38^) are dwarfed by the 5000 fold increase observed here. Pleural fluid IL-6 is associated with both radiological markers and increased hospital length of stay; in addition to contributing to our understanding of pleural infection pathophysiology, this provides a compelling target for immunomodulation.

Previous attempts to immunomodulate in pleural infection have focused on steroids. The Steroid Therapy and Outcome of Parapneumonic Pleural Effusions (STOPPE) Trial randomised patients with community-acquired pneumonia and associated pleural effusions to intravenous dexamethasone (4 mg twice daily for 48 h) or placebo.^7^ Whilst no effect was seen in terms of improvement in clinical outcomes, the study was relatively small (n = 80) and included patients with small parapneumonic effusions through to frank empyema. A similar randomised trial was carried out with children presenting with an effusion secondary to pneumonia.^8^ 60 patients were randomised to intravenous dexamethasone or placebo. The trial was too small to draw definitive conclusions, but there was a suggestion of shorter time to recovery in the intervention group (109 h vs. 177 h, p = 0.04).

Our observational study was small, and as such precision was limited. We cannot therefore be confident about the role of IL-6 in predicting severe consequences of pleural infection, although it is likely that our findings of increased IL-6 lie on the causal path to the more severe outcomes in pleural infection.

For our genetic study, our limitations are that of all MR analyses: we cannot test all the assumptions of MR, and it is plausible that our analyses are biased by genetic confounding or other related issues. Against that, *cis* MR at the IL6R gene is one of the most established techniques in MR,^22^ and MR findings correlate with RCT data at this locus well. One other key limitation is that we use a case–control study of pleural infection incidence as our outcome GWAS. That is, our MR findings should be interpreted as showing that IL-6 activity is causally predicted to increase the odds of development of infection, and not that IL-6 activity during an episode of infection is thereby pathological. However, the same findings on incidence of severe COVID-19 were used successfully as evidence for IL-6 inhibition in severe COVID-19, so we can have some confidence that our genetic findings might translate therapeutically. Finally, the genetic analyses in this study were restricted to individuals of European ancestry due to the source of the available GWAS summary statistics (primarily UK Biobank and FinnGen). This significantly limits the generalisability of the MR findings to individuals of African, Asian, or other non-European ancestries, where allele frequencies, LD patterns, and gene–environment interactions may differ. Future research leveraging emerging large-scale biobanks with greater ancestral diversity, such as the Million Veteran Program,^40^ will be crucial to assess the role of IL-6R signalling in pleural infection across diverse populations, once adequately powered GWAS for both IL-6R/CRP and relevant infection outcomes become available in these cohorts. The use of exposure and outcome GWAS studies that both contained individuals from the UK Biobank also contributed to a potential sample overlap of up to 6.5%, however as demonstrated in simulation studies this is unlikely to be significant given the strength of IVs used in this analysis.^41^

Our data supports a randomised trial of IL-6 inhibition in pleural infection. Questions remain about how this could be conducted. Firstly, should the target population include patients with parapneumonic effusion at risk of developing pleural infection, or those who already have a diagnosis of pleural infection? Secondly, how should IL-6 be inhibited, and by which route? Given likely reduced systemic effect with intrapleural administration, and the stronger relationship between pleural IL-6 and outcomes, intrapleural may be favoured over systemic administration. Thirdly, are there any risks of making pleural infection worse by inhibiting IL-6? Data from COVID-19 trials are reassuring here, showing no increase in bacterial superinfection in those assigned to IL-6 inhibition, but the pathophysiology is very different.^12^

In this study we have shown that, in patients with pleural infection, IL-6 is significantly raised in pleural fluid compared to serum, suggesting a local inflammatory response. Pleural fluid IL-6 can predict hospital length of stay and correlates with other important markers of severity. There is evidence to suggest that IL-6 downregulation reduces the incidence of pleural infection and represents a promising target for future trials.

## Contributors

FH and DA devised the study. AA and KE performed the laboratory work, while AR performed the genetic analyses with support of HW, RB and MW, and supervision from FH, HW and RB. MW provided critical appraisal of the manuscript. NM and DA provided clinical expertise. AA and DA had access to and verified the observational study data. AR and FH had access to and verified the MR data. All authors were involved in the writing and reviewing, and have approved the final manuscript.

## Data sharing statement

The data that support the findings of this study are provided within the Article and its appendix. Our work makes use of study-level summary statistics. We report summary statistics in the GitHub repository (https://github.com/gushamilton/empyema_il6) to permit independent interrogation of our analyses. Further requests for data or other relevant information can be directed to the corresponding authors or members of the Bristol Academic Respiratory Unit.

## Declaration of interests

The authors report no conflicts of interest.
